# Abundance of water bodies is critical to guide mosquito larval control interventions and predict risk of mosquito-borne diseases

**DOI:** 10.1186/1756-3305-6-179

**Published:** 2013-06-18

**Authors:** Denis Valle, Benjamin Zaitchik, Beth Feingold, Keith Spangler, William Pan

**Affiliations:** 1 University Program in Ecology, Duke University, Durham, NC, 27708, USA; 2 Department of Earth and Planetary Sciences, Johns Hopkins University, Baltimore, MD, 21218, USA; 3 Nicholas School of the Environment and the Duke Global Health Initiative, Duke University, Durham, NC, 27708, USA

**Keywords:** Mosquito breeding habitat, Larval control, Malaria, Disease risk mapping, Distribution of water bodies, *Anopheles*

## Abstract

Characterization of mosquito breeding habitats is often accomplished with the goal of guiding larval control interventions as well as the goal of identifying areas with higher disease risk. This characterization often relies on statistical measures of association (e.g., regression coefficients) between covariates and presence/absence or abundance of larva. Here we contend that these measures of association are not enough; researchers should also study the spatial and temporal distribution of water bodies. We provide recommendations on how current methodology may be improved to adequately take into account the distribution of water bodies.

## Letter to the editor

Studies on mosquito breeding sites typically survey water bodies to determine larval presence or abundance. Then, measures of association are estimated (e.g., regression coefficients, correlation coefficients, or analysis of variance) and used to identify important predictors of larval presence, with the goal of guiding larval control interventions and predicting disease risk. A small sample of entomological studies that follow this generic recipe is given in the Additional file 1. While these measures of association are important to characterize larval habitat, here we contend that these measures may not be enough to guide larval control initiatives and determine disease risk.

Our contention is based on the same arguments as those that motivated the creation of the population attributable fraction (PAF) concept. In the case of PAF, it has been argued that measures of association do not take into account the prevalence of the different risk factors. Thus, a particular risk factor might be statistically significant but have small public health relevance if very few people have that risk factor [1]. Similarly, the risk factors associated with very productive larval habitats (defined here as a water body that typically has larvae) might not be relevant for larval control if water bodies with those risk factors are rare in the overall landscape.

Determining the relative abundance of water bodies is also critical when predicting disease risk. Researchers often perform their analysis given that a water body was sampled. In statistical terms, the typical analysis makes inference on the conditional probability *p*(*L*|*W*), where L and W denote the presence of larva and the event that a water body was sampled, respectively. On the other hand, to understand disease risk, inference should be made on the marginal probability *p*(*L*).

To illustrate, consider the simplified example summarized in Table 1. Water bodies are sampled in a forested and a deforested site using the same number of transects per site. In scenario 1, these transects yield 30 water bodies (8 of which had larvae) in the forested sites and 10 water bodies (8 of which had larvae) in the deforested site. As a result, the proportion of water bodies with larvae is $p_{\mathrm{for}}⌢=8/30\approx 0.27$ and $p_{\mathrm{def}}⌢=8/10=0.8$, for the forested and deforested sites, respectively. Based on these probabilities, a logistic regression would indicate that forest cover is negatively associated with the presence of the mosquito larvae and a researcher would conclude that people living at forested sites have a lower infection risk. This conclusion is incorrect, since both sites have the same number (i.e., 8) of water bodies with larvae per area. If these sites give rise to a similar number of larvae and adult mosquitoes, and if these mosquitoes have the same degree of contact with the host, then infection risk should be similar. Alternatively, scenario 2 assumes that the proportion of water bodies with larvae is identical in both sites $p_{\mathrm{for}}⌢=p_{\mathrm{def}}⌢=1/2$ and a logistic regression would fail to find significant differences between sites, despite the fact that the forested site has three times more water bodies with larvae per area when compared to the deforested site. Although over-simplistic, these examples are useful to highlight that it is critical to identify the characteristics of productive larval sites and to take into account the prevalence of water bodies with these characteristics. For completeness, we provide examples with simulated and real data in the Additional file 1.

**Table 1:** Description of outcomes for scenarios 1 and 2

**Outcomes**	**Scenario 1**		**Scenario 2**	
	**Forested**	**Deforested**	**Forested**	**Deforested**
# of water bodies	30	10	30	10
# of water bodies with larva	8	8	15	5
Proportion of water bodies with larva	0.3	0.8	0.5	0.5

We believe it is important for researchers to carefully consider how the outcome of their analysis could inform policy actions. We re-iterate that the typical regression analysis assumes water bodies to be the sampling unit, thus yielding results per water body. If the researcher is primarily interested in infection risk, however, it is likely that the response variable more closely associated with infection risk is in areal unit (e.g., number of water bodies with larvae per transect). In other words, there is a mismatch between the analyzed outcome and the outcome more relevant for public health policy making. To avoid this mismatch, we propose two alternatives. First, one can directly model the number of water bodies with larvae per transect as a function of transect-level covariates, assuming that the sampling unit is the transect itself. Alternatively, one can predict the number of water bodies per transect to then predict how many have larvae (as in Additional file 1: Figure S2). Both approaches could also be used for fixed-area plots. In either modeling approach, the sampling design for water bodies is critical and merits careful consideration. Unfortunately, most studies provide detailed descriptions on how larvae were sampled within water bodies but not how water bodies themselves were sampled (e.g. [2]).

We emphasize that using water bodies as the sampling unit is perfectly valid to characterize larval habitat. However, researchers should be careful when using the derived measures of association to identify larval control strategies and predict disease risk. While dengue researchers have long recognized the importance of accounting for the abundance of water containers (e.g. [3]), we believe that the issues we raise have not been taken into account for other vector-borne diseases. Although we have focused on mosquito larval habitat, our results are likely to apply to other types of disease vectors that also rely on water bodies.

## Competing interests

The authors declare that they have no competing interests.

## Authors’ contributions

DV analyzed the simulated and real datasets and drafted the manuscript. BZ and KS generated the land use / land cover and climate covariates. WP, BF, KS, and BZ revised the manuscript and suggested improvements. All authors read and approved the final version of the manuscript.

## Supplementary Material

Additional file 1 Sample of studies on mosquito breeding habitat, examples with real and simulated data, and description of the model used to analyze Anopheles darlingi data. Click here for file
