# Supplementary material for: Abundance of water bodies is critical to guide mosquito larval control interventions and predict risk of mosquito-borne diseases
Source: Parasit Vectors. 2013 Jun 18;6:179. doi: 10.1186/1756-3305-6-179 (PMC3698051; doi:10.1186/1756-3305-6-179)
Supplement: Additional file 1 — Sample of studies on mosquito breeding habitat, examples with real and simulated data, and description of the model used to analyze Anopheles darlingi data. [file 1756-3305-6-179-S1.docx]

## Additional file

## Sample of studies that follow the generic recipe described in the main text

Table S1: Sample of studies on mosquito breeding habitat.

| Vector | Disease | Country | Source |
| --- | --- | --- | --- |
| *Anopheles gambiae* | Malaria | Kenya | [[1](#_ENREF_1)] |
| *Anopheles* sp. | Malaria | Kenya | [[2](#_ENREF_2)] |
| *Culex* | Filariasis and Arboviruses | Kenya | [[3](#_ENREF_3)] |
| *Anopheles* sp. | Malaria | Kenya | [[4](#_ENREF_4)] |
| *Anopheles* sp. | Malaria | Ethiopia | [[5](#_ENREF_5)] |
| *A. gambiae* | Malaria | Ghana | [[6](#_ENREF_6)] |
| *Anopheles* sp. | Malaria | Tanzania | [[7](#_ENREF_7)] |
| *Anopheles* sp. | Malaria | Côte d’Ivoire | [[8](#_ENREF_8)] |
| *Anopheles darlingi* | Malaria | Peru | [[9](#_ENREF_9)] |
| *Anopheles sinensis* | Malaria | China | [[10](#_ENREF_10)] |
| *Aedes* sp. */ Anopheles* sp. | Dengue / Malaria | Thailand | [[11](#_ENREF_11)] |
| *Aedes aegypti* | Yellow fever / Dengue | Argentina | [[12](#_ENREF_12)] |

## Simulation results

Our simulation assumes that we have 25 transects in each area, which are either forested or deforested. Let $W_{i}$ be the number of water bodies for transect *i (i=1,…,25)*, where $E[W_{i}]=\lambda$, and $p$be the proportion of water bodies with larva. We assume that the forested area has an average of 30 water bodies per transect ($E\left[ W_{i}^{for} \right]=\lambda^{for}=30$), from which an average 27% have larva ($p^{for}=0.2\bar{6}$), while the deforested area tends to have less water bodies on average per transect ($E\left[ W_{i}^{def} \right]=\lambda^{def}=10$) but a greater proportion of them tend to have larva ($p^{def}=0.8$). Finally, let the number of water bodies with larva for transect *i* be denoted as $L_{i}^{for}$ and $L_{i}^{def}$, for the forested and deforested sites, respectively.

In our simulations, we assume that:

$$W_{i}^{for}|\lambda^{for}\sim Poisson(\lambda^{for})$$

$$W_{i}^{def}|\lambda^{def}\sim Poisson(\lambda^{def})$$

With these assumptions, it is trivial to show that the expected number of water bodies with larva per transect is the same for the forested and deforested sites (i.e., $E[L_{i}^{j}]=E[E[L_{i}^{j}|W_{i}^{j}]]=E[W_{i}^{j}p^{j}]=\lambda^{j}p^{j}=8$, where $j=def$ or $j=for$). Using data generated in this fashion, we can run a binomial regression to show that there is a significant difference between deforested and forested areas on the proportion of water bodies with larva, despite the same average number of water bodies with larva per transect.

Alternatively, we can consider the abundance of larva per transect instead of the number of water bodies with larva per transect. Let the average larva abundance per water body be denoted by $\delta$ and $L_{i}$ be the number of larvae for water body *i*. We assume that the forested area has an average larva abundance per water body of 3.3 ($\delta^{for}=3.\bar{3}$) whereas the deforested area has a higher average larva abundance per water body ($\delta^{def}=10$). We now assume that:

$$W^{for}|\lambda^{for}\sim Poisson(\lambda^{for})$$

$$W^{def}|\lambda^{def}\sim Poisson(\lambda^{def})$$

$L_{i}^{for}|\delta^{for}\sim Poisson(\delta^{for})$ for each water body

$L_{i}^{def}|\delta^{def}\sim Poisson(\delta^{def})$ for each water body

Again, the expected number of larva per transect is the same for the forested and deforested areas (i.e., $E[\sum_{i=1}^{W^{j}} L_{i}^{j}]=E[E[\sum_{i=1}^{W^{j}} L_{i}^{j}|W^{j}]]=E[W^{j}\delta^{j}]=\lambda^{j}\delta^{j}=100$, where *j=def* or *j=for*). Using these data, we can run a Poisson regression to show that there is a significant difference between the forested and deforested site in terms of the larva abundance per water body, despite the same average larva abundance per transect.

We performed 1000 simulations and show that both the binomial and the Poisson regressions consistently estimate a statistically significant difference between forested and deforested sites (upper panels in Figure S1), despite the fact that these datasets were created so that both sites have the same average number of water bodies with larva or larva abundance per transect (lower panels in ).


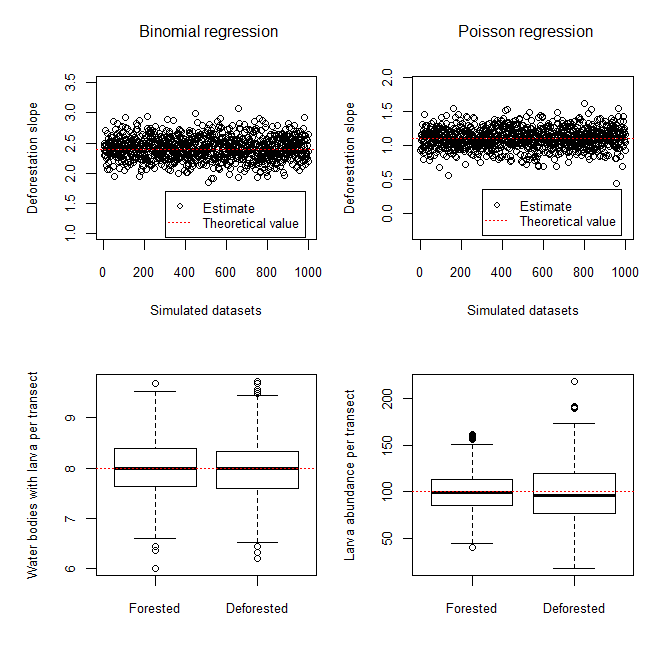


Figure S1: Regression models detect a significant difference between forested and deforested sites (upper panels), despite the same average per transect (lower panels).

Simulation results show that Binomial (upper left panels) and Poisson (upper right panels) regressions can indicate a significant difference between the forested and deforested sites even if the average number of water bodies with larva (lower left panel) or larva abundance (lower right panel) per transect is the same for both sites.

## Example with real data

To illustrate this problem with real data, we estimated how the proportion of water bodies with larva changes with forest cover for *Anopheles darlingi*, the primary malaria vector in the Amazon region, using existing data from the Peruvian Amazon. Details regarding how data were collected are available in the original paper [[9](#_ENREF_9)]. We fitted a binomial model (model details are given below), which revealed that the probability of a water body having *A. darlingi* larvae decreases with forest cover (upper panel in ). However, depending on the relationship between the number of water bodies per transect and forest cover (middle panels in ), the relationship between the number of water bodies with larva per transect and forest cover can vary substantially and even reverse sign (lower panels in ), fundamentally changing the resulting inference on the effect of deforestation.


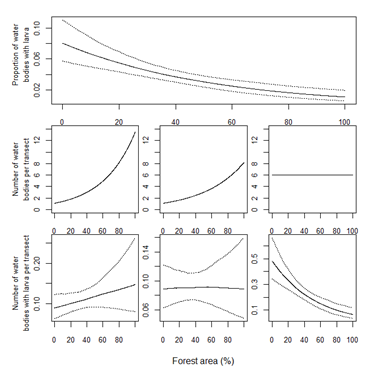


Figure S2: The number of water bodies with larva per transect (lower panels) is influenced by the relationship between the proportion of water bodies with larva and forest area (upper panel) *and* the relationship between number of water bodies per transect and forest area (middle panels).

Solid and dashed lines are median and 95% credible intervals, respectively.

## Description of the binomial model used to fit the A. darlingi larva data.

### Data

We surveyed larval anophelines in 56 locations (i.e., 14 sites x 4 vegetation types within each site). Larvae were collected once every three weeks between March and August 2001 (resulting in 8 collections) from water bodies found along multiple transects. Further details regarding the larval collection methodology can be found in [[9](#_ENREF_9),[13](#_ENREF_13)].

Larva abundance data greatly depends on methodological details (e.g., where and how samples were taken as well as how many larvae died before being identified), resulting in considerable variability. To avoid this variability, we converted these data to presence/absence data. Data are summarized for each location *j* (j=1,…,56) and collection *t* (t=1,…,8) as the number of water bodies with larva $L_{jt}$ and the total number of sampled water bodies $W_{jt}$.

### Covariates

The covariates we used were land use / land cover (LULC) and climate related covariates, estimated from satellite data. The set of LULC variables includes urban, water, forest, and non-forest vegetation areas, estimated by conducting a supervised classification of a 2001 Landsat image within a radius of 1000 m from the center of each larva transect.

The climate covariates include precipitation and solar radiation at the day of collection and average (over the last 5 days prior to the collection date) minimum temperature, solar radiation and soil moisture. Solar radiation and minimum daily temperature were drawn from the Global Data Assimilation System [[GDAS; 14, 0.471 degree resolution](#_ENREF_14)] gridded analysis and were topographically downscaled to 1km resolution using standard lapse rate corrections [[e.g., 15](#_ENREF_15)]. Precipitation estimates are from the Tropical Rainfall Measurement Mission (TRMM) Multisensor Precipitation Analysis [[TMPA; 16](#_ENREF_16)]. The three hourly, 25km gauge corrected estimates were used (product 3B42v7). GDAS meteorological fields and TRMM precipitation were applied as forcing data to offline simulations with the Noah Land Surface Model v3.2 [[17](#_ENREF_17),[18](#_ENREF_18)], implemented in the NASA Land Information System [[19](#_ENREF_19)]. Noah simulations were used to generate estimates of surface and root zone soil moisture every three hours over the period of analysis.

### Methods

We employ a binomial model

,

where $p_{jt}$ is assumed to be a function of our covariates

$\text{logit}(p_{jt})=\alpha+\mathbf{x}_{\mathbf{jt}}^{\mathbf{T}}\mathbf{\beta}$ .

We adopt vague priors for the intercept $\alpha$ but multiple shrinkage prior for the slope parameters $\mathbf{\beta}$, which allows for priors to be stronger or weaker depending on the magnitude of the slope parameter:

$\alpha\sim N(0,10)$ ,

$\beta_{k}|\tau_{k}^{2}\sim N(0,\tau_{k}^{2})$,

$\tau_{k}^{2}|\lambda\sim Exponential(\lambda)$ .

We chose $E[\tau_{k}^{2}]=1/\lambda=0.01$ which implies $\lambda=100$. This assumes that the prior on the slope parameter will have approximately an average standard deviation of 0.1.

This model was fitted using a Gibbs sampler, with Metropolis-within-Gibbs steps to sample the full conditionals. On total, 100,000 iterations were run and the first 10,000 iterations were discarded as burn-in. Convergence was assessed through trace-plots. All analyzes and plots were created in R [[20](#_ENREF_20)].

### Model fit and regression coefficients

The comparison of the posterior predictive distribution and the larva data suggests an adequate fit of our model ().


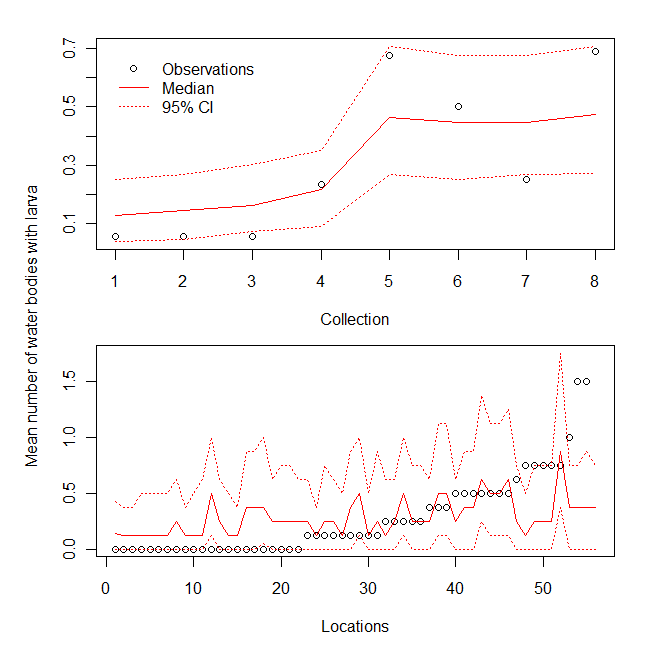


Figure S3: Comparison of the predicted and observed average number of water bodies with *Anopheles darlingi* larva, for different collections periods (upper panel) and locations (lower panel).

The posterior distribution of the slope parameters suggest that forest area and average minimum temperature, solar radiation and soil moisture, significantly influence the probability of finding *A. darlingi* larvae in water bodies ().


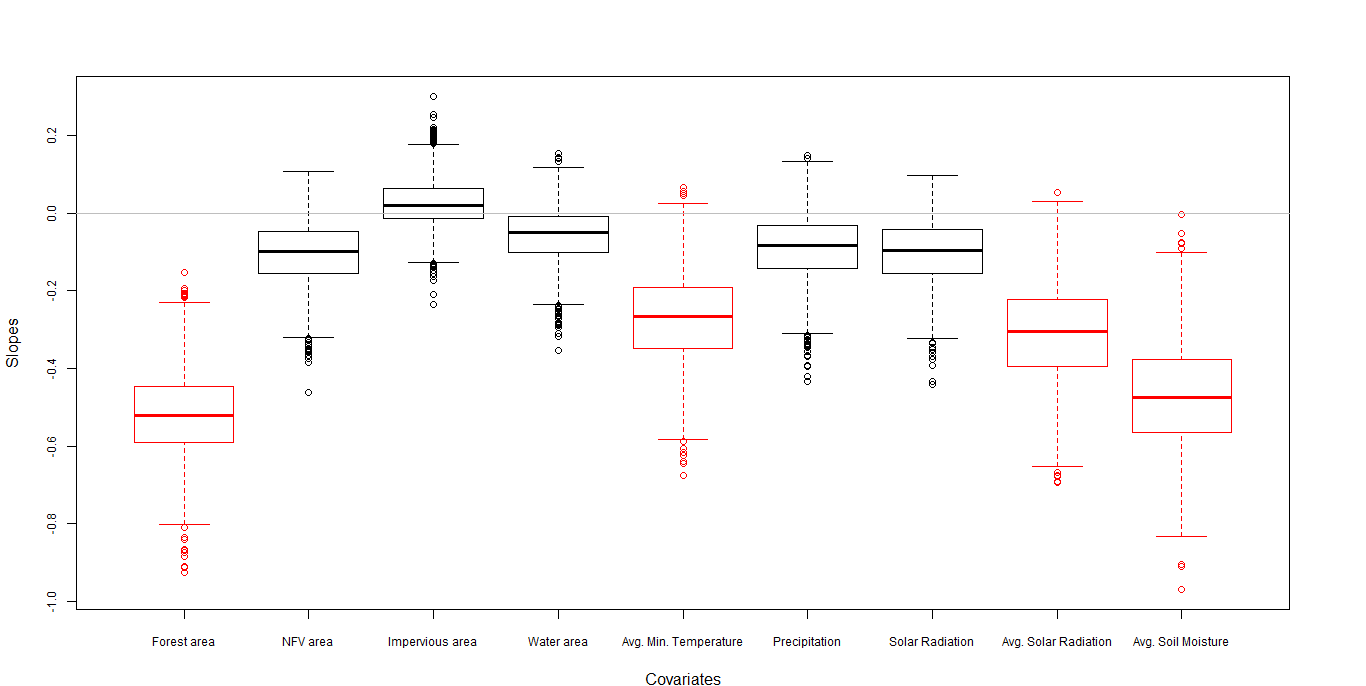


Figure S4: Posterior distribution of slope parameters.

Red boxes indicate slopes that are significantly different from zero. NFV stands for non-forest vegetation area and Avg. denotes average for 5-days prior to the collection date.

References

1. Mala AO, Irungu LW, Shililu JI, Muturi EJ, Mbogo CC, et al. (2011) Dry season ecology of Anopheles gambiae complex mosquitoes at larval habitats in two traditionally semi-arid villages in Baringo, Kenya. Parasit Vectors 4 (25).

2. Mwangangi JM, Shililu JI, Muturi EJ, Muriu S, Jacob B, et al. (2010) Anopheles larval abundance and diversity in three rice agro-village complexes Mwea irrigation scheme, central Kenya. Malar J 9 (228).

3. Muturi EJ, Shililu JI, Gu W, Jacob BG, Githure JI, et al. (2007) Larval habitat dynamics and diversity of Culex mosquitoes in rice agro-ecosystem in Mwea, Kenya. Am J Trop Med Hyg 76 (1): 95-102.

4. Ndenga BA, Simbauni JA, Mbugi JP, Githeko AK (2012) Physical, chemical and biological characteristics in habitats of high and low presence of Anopheline larvae in Western Kenya Highlands. PLOS One 7 (10): e4797.

5. Kenea O, Balkew M, Gebre-Michael T (2011) Environmental factors associated with larval habitats of anopheline mosquitoes (Diptera: Culicidae) in irrigation and major drainage areas in the middle course of the Rift Valley, central Ethiopia. Journal of Vector Borne Disease 48: 85-92.

6. Afrane YA, Lawson BW, Brenya R, Kruppa T, Yan G (2012) The ecology of mosquitoes in an irrigated vegetable farm in Kumasi, Ghana: abundance, productivity and survivorship. Parasit Vectors 5 (233).

7. Sattler MA, Mtasiwa D, Kiama M, Premji Z, Tanner M, et al. (2005) Habitat characterization and spatial distribution of Anopheles sp. mosquito larvae in Dar es Salaam (Tanzania) during an extended dry period. Malar J 4 (4).

8. Matthys B, N'Goran EK, Kone M, Koudou BG, Vounatsou P, et al. (2006) Urban agricultural land use and characterization of mosquito larval habitats in a medium-sized town of Cote d'Ivoire. J Vector Ecol 31 (2): 319-333.

9. Vittor AY, Pan W, Gilman RH, Tielsch J, Glass G, et al. (2009) Linking deforestation to malaria in the Amazon: characterization of the breeding habitat of the principal malaria vector, Anopheles darlingi. Am J Trop Med Hyg 81 (1): 5-12.

10. Liu X-B, Liu Q-Y, Guo Y-H, Jiang J-Y, Ren D-S, et al. (2012) Random repeated cross sectional study on breeding site characterization of Anopheles sinensis larvae in distinct villages of Yongcheng City, People's Republic of China. Parasit Vectors 5 (58).

11. Vanwambeke SO, Somboon P, Harbach RE, Isenstadt M, Lambin EF, et al. (2007) Landscape and land cover factors influence the presence of Aedes and Anopheles larvae. J Med Entomol 44 (1): 133-144.

12. Vezzani D, Rubio A, Velazquez SM, Schweigmann N, Wiegand T (2005) Detailed assessment of microhabitat suitability for Aedes aegypti (Diptera: Culicidae) in Buenos Aires, Argentina. Acta Trop 95: 123-131.

13. Vittor AY (2003) Deforestation and malaria: associations between vegetation, vector ecology and malaria epidemiology in the Peruvian Amazon. Baltimore, Maryland: Johns Hopkins University.

14. Derber JC, Parrish DF, Lord SJ (1991) The New Global Operational Analysis System at the National-Meteorological-Center. Weather Forecast 6: 538-547.

15. Liston GE, Elder K (2006) A Meteorological Distribution System for High-Resolution Terrestrial Modeling (MicroMet). J Hydrometeorol 7: 217-234.

16. Huffman GJ, Adler RF, Bolvin DT, Gu GJ, Nelkin EJ, et al. (2007) The TRMM multisatellite precipitation analysis (TMPA): Quasi-global, multiyear, combined-sensor precipitation estimates at fine scales. J Hydrometeorol 8: 38-55.

17. Chen F, Mitchell K, Schaake J, Xue YK, Pan HL, et al. (1996) Modeling of land surface evaporation by four schemes and comparison with FIFE observations. J Geophys Res-Oc Atm 101: 7251-7268.

18. Ek MB, Mitchell KE, Lin Y, Rogers E, Grunmann P, et al. (2003) Implementation of Noah land surface model advances in the National Centers for Environmental Prediction operational mesoscale Eta model. J Geophys Res-Oc Atm 108.

19. Kumar SV, Peters-Lidard CD, Tian Y, Houser PR, Geiger J, et al. (2006) Land information system: An interoperable framework for high resolution land surface modeling. Environ Modell Softw 21: 1402-1415.

20. R Development Core Team (2010) R: A language and environment for statistical computing. Vienna, Austria: R Foundation for Statistical Computing. <http://www.R-project.org>
